# RELATIONSHIPS BETWEEN CEREBRAL VASCULOPATHIES AND MICROINFARCTS IN COMMUNITY-BASED OLDER ADULTS

**DOI:** 10.1093/geroni/igad104.1272

**Published:** 2023-12-21

**Authors:** Mo-kyung Sin, Yan Cheng, Jeffrey Roseman, Edward Zamrini, Ali Ahmed

**Affiliations:** Seattle University, Seattle, Washington, United States; George Washington University, Washington, District of Columbia, United States; University of Alabama at Birmingham, Birmingham, Alabama, United States; Irvine Clinical Research, Irvine, California, United States; Washington DC Veterans Medical Center, Washington, District of Columbia, United States

## Abstract

Cerebral microinfarcts are associated with cognitive impairment and dementia. Less is known about the relationships of various vasculopathies (arteriolosclerosis and cerebral amyloid angiopathy (CAA) with microinfarcts. This case-control study examined the associations of the vasculopathies with microinfarcts in 842 older adults in the Adult Changes in Thought (ACT) autopsy data. Arteriolosclerosis was assessed by histological examination using the H&E stain; CAA was assessed using the Congo red histochemical stains; and microinfarcts was ascertained by blinded board-certified neuropathologists. Multivariable-adjusted logistic regression models were used to estimate odds ratios (ORs) and 95% confidence intervals (CI) for the presence of cerebral microinfarcts associated with the two cerebral vasculopathies. Cerebral microinfarcts were present in 417 (49.5%) participants. Mild, moderate, and severe arteriolosclerosis was present in 13.2%, 43.9%, 29.7% of participants with microinfarcts, and 28.9%, 40.0%, and 12.5% of participants without microinfarcts, respectively. ORs (95% CIs) for microinfarcts associated with moderate and severe arteriolosclerosis were 2.15 (1.45-3.17) and 4.59 (2.88-7.33), respectively. Mild, moderate, and severe CAA was present in 18.0%, 17.5%, and 3.6% of participants with microinfarcts, and 18.1%, 16.5%, and 2.4% of participants without microinfarcts, respectively. ORs (95% CIs) for microinfarcts associated with moderate and severe CAA were 0.97 (0.65-1.44) and 1.37 (0.59-3.21), respectively. Cerebral microinfarcts were common in older adults and were associated with a higher prevalence of arteriolosclerosis, but not with CAA. Our study underscores the need for additional studies to better understand this complex relationship to develop interventions for primary prevention of cognitive impairment.

